# Extracellular plant subtilases dampen cold-shock peptide elicitor levels

**DOI:** 10.1038/s41477-024-01815-8

**Published:** 2024-10-11

**Authors:** Changlong Chen, Pierre Buscaill, Nattapong Sanguankiattichai, Jie Huang, Farnusch Kaschani, Markus Kaiser, Renier A. L. van der Hoorn

**Affiliations:** 1 Institute of Biotechnology, Beijing Academy of Agriculture and Forestry Sciences, Beijing, China; 2 Plant Chemetics Laboratory, Department of Biology, University of Oxford, Oxford, UK; 3 ZMB Chemical Biology, Faculty of Biology, University of Duisburg-Essen, Essen, Germany

**Keywords:** Pattern recognition receptors in plants, Biotic, Microbe

## Abstract

Recognizing pathogen-associated molecular patterns on the cell surface is crucial for plant immunity. The proteinaceous nature of many of these patterns suggests that secreted proteases play important roles in their formation and stability. Here we demonstrate that the apoplastic subtilase SBT5.2a inactivates the immunogenicity of cold-shock proteins (CSPs) of the bacterial plant pathogen *Pseudomonas syringae* by cleaving within the immunogenic csp22 epitope. Consequently, mutant plants lacking SBT5.2a activity retain higher levels of csp22, leading to enhanced immune responses and reduced pathogen growth. SBT5.2 sensitivity is influenced by sequence variation surrounding the cleavage site and probably extends to CSPs from other bacterial species. These findings suggest that variations in csp22 stability among bacterial pathogens are a crucial factor in plant–bacteria interactions and that pathogens exploit plant proteases to avoid pattern recognition.

## Abstract (web summary)

Secreted plant subtilase SBT5.2 inactivates immunogenic csp22 epitopes in cold-shock proteins of *Pseudomonas syringae*, suggesting that pathogens exploit plant proteases to evade detection.

## Main

Pathogen recognition is pivotal to plant survival. Pathogen-associated molecular patterns (PAMPs) are recognized on the plant cell surface by pattern-recognition receptors (PRRs). Upon PAMP binding, PRRs associate with receptor kinases and intracellular protein kinases to activate pattern-triggered immunity (PTI), which includes the production of reactive oxygen species (ROS) and ethylene, apoplast alkalinization, MAP kinase phosphorylation and transcriptional reprogramming^1–3^.

PAMP recognition by PRRs occurs in the extracellular space within plant tissues (the apoplast). The apoplast is the first and often final destination for pathogens and is an important site for pathogen proliferation^3^. Pathogen colonization of the apoplast is partly mitigated by plant-secreted hydrolases, including proteases, which are secreted both constitutively and inducibly^4,5^. Ser proteases, including subtilases, are the largest class of secreted proteases^4^.

PAMPs can be oligosaccharides, lipids and peptides, and a wide range of peptide-based PAMPs have been identified from fungal, oomycete and bacterial pathogens^6^. PRRs tend to perceive a conserved epitope of a PAMP that has important functions to the microorganism^7^. Although the release of immunogenic PAMP peptides from their precursors could be an essential step in pathogen recognition, our knowledge of the biogenesis and maintenance of PAMPs and the involved enzymes is very limited^8^.

The conserved nucleic acid binding motif RNP-1 of bacterial cold-shock proteins (CSPs) serves as a PAMP that is perceived by PRRs from several Solanaceae species, including tomato (*Solanum lycopersicum*), tobacco (*Nicotiana tabacum*) and potato (*S. tuberosum*)^9–11^. CSPs are highly induced in bacteria in response to rapid downshifts in temperature, which is the basis for their name. However, CSPs are also activated by other types of stress, and many are constitutively produced^12^. The 22-residue amino-terminal consensus of 150 bacterial CSP sequences from genera such as *Micrococcus*, *Bacillus* and *Escherichia* was introduced as csp22. Residues 5–19 of csp22 (csp15) constitute the active PAMP epitope that can trigger plant immune responses, including a burst of ROS^9,10^. Two distinct receptors, the receptor-like protein CSPR and the receptor-like kinase CORE, were claimed to act as CSP receptors in older *Nicotiana* *benthamiana* plants^10,11^, but the affinity of csp22 for CORE is much higher than that for CSPR^11^, and only the silencing of *CORE* makes these plants insensitive to csp22 (ref. ^13^). CSPR was later identified as RE02 and is involved in perceiving small Cys-rich proteins from *Valsa mali*^14^ and *Sclerotinia sclerotiorum*^15^.

In this study, we tested the hypothesis that extracellular plant proteases might play important roles in the biogenesis of the CSP elicitor of the model bacterial pathogen *Pseudomonas syringae* pv. *tomato* DC3000 (*Pto*DC3000). Surprisingly, however, we discovered proteolytic degradation of csp22 in the apoplast, mediated by serine protease SBT5.2, which dampens the CSP-triggered immune response and suggests that pathogens take advantage of plant proteases to escape PAMP recognition.

## Results

### csp22 from CspD of *Pto*DC3000 is recognized by CORE

**Fig. 1:**
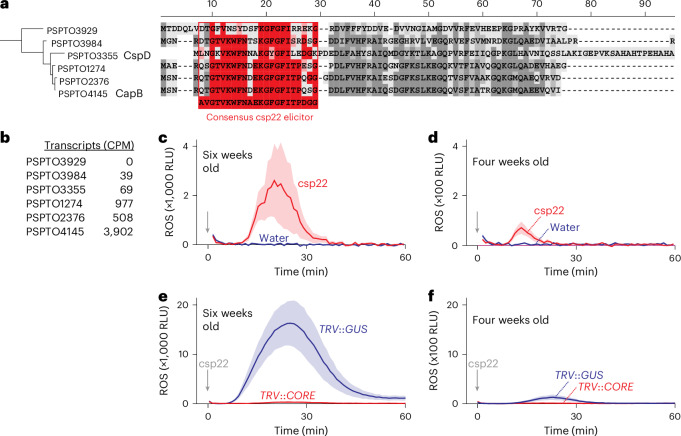
CspD-derived csp22 triggers *Nb*CORE-dependent oxidative burst in *N. benthamiana.* **a**, Sequence alignment of the six CSPs of *Pto*DC3000 with the frequently used consensus csp22 peptide. **b**, Transcript levels of CSPs of *Pto*DC3000 during infection in counts per million (CPM). Extracted from ref. ^16^. **c**, CspD-derived csp22 triggers an oxidative burst in six-week-old *N. benthamiana* plants. **d**, CspD-derived csp22 triggers only a weak oxidative burst in four-week-old *N. benthamiana* plants. **e**, The oxidative burst triggered by csp22 of CspD in six-week-old plants is absent when *NbCORE* is silenced. **f**, The weak oxidative burst triggered by csp22 of CspD in four-week-old plants is absent when *NbCORE* is silenced. In **c**–**f**, one-week-old *N. benthamiana* plants were infected with and without TRV carrying a fragment of *GUS* (*TRV*::*GUS*) or *NbCORE* (*TRV*::*CORE*). Leaf discs of four- and six-week-old plants were treated with water or 500 nM csp22, and ROS were measured in relative light units (RLU). The lines represent the mean and the shading represents the s.e.m. of *n* = 12 (**c**,**d**) or *n* = 6 (**e**,**f**) leaf discs.

**Extended Data Fig. 1:**
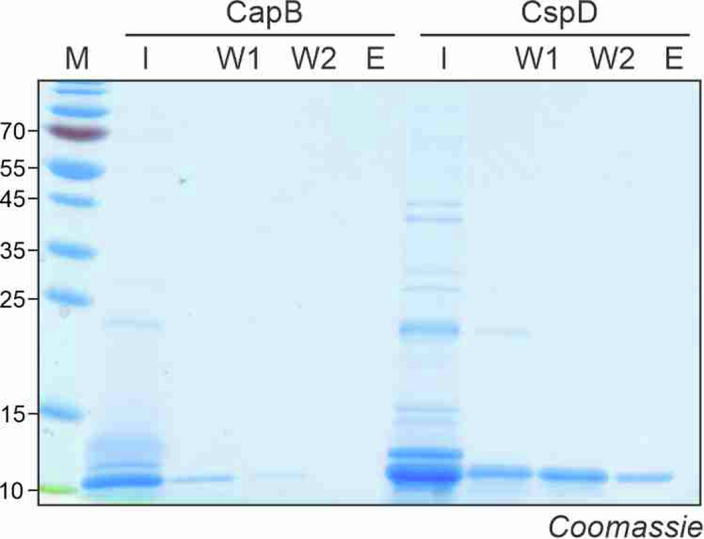
Expression and purification of CapB and CspD. CapB and CspD with expressed with N-terminal His-TEV peptides in E. coli and purified over Ni-NTA. I, input sample; W1, wash with 100 mM imidazole; W2, wash with 150 mM imidazole; E, eluate with 250 mM imidazole. Samples were separated by SDS-PAGE and stained with Coomassie. This experiment was repeated two times with similar results.

To determine the role of CSP elicitor release in bacterial immunity, we studied the release of csp22 from *Pto*DC3000. The *Pto*DC3000 genome encodes six CSPs that contain the cold-shock domain (PF00313) and comprehend peptides similar to the csp22 consensus (Fig. 1a). Five of these six CSP-encoding genes are expressed during infection^16^ (Fig. 1b). Since the original discovery of csp22 included the CspD and CapB proteins from various bacteria^9^, we expressed these two proteins in *Escherichia coli* with a His tag linked via the TEV cleavage sequence to the N terminus. Heterologous expression only delivered sufficient CspD protein (Extended Data Fig. 1), so we continued our studies with CspD.

The csp22 peptide of CspD differs at nine residues from the consensus csp22 reported originally^9^. To test whether the csp22 peptide of CspD is recognized by *N. benthamiana*, we synthesized this 22-residue peptide and added it to leaf discs of six-week-old *N. benthamiana* floating on a solution containing luminol and horseradish peroxidase (HRP). Luminescence measurements revealed that csp22 of CspD is able to trigger a classic oxidative burst by releasing ROS, in contrast to the water control (Fig. 1c). Only a weak oxidative burst was detected in four-week-old plants (Fig. 1d), consistent with the low expression of *Nb*CORE in younger plants^11^.

To confirm that *Nb*CORE is required for detecting csp22 of CspD, we depleted *Nb*CORE with virus-induced gene silencing (VIGS) using tobacco rattle virus (TRV) vectors carrying a 300-bp fragment of *Nb*CORE, or of β-glucuronidase (GUS) as a negative control^13^. Oxidative burst assays showed that six-week-old *TRV*::*CORE* plants are blind to csp22 of CspD, in contrast to *TRV*::*GUS* plants, which show a csp22-induced oxidative burst (Fig. 1e). The weak csp22-induced response detected in four-week-old *TRV*::*GUS* plants is also absent from *TRV*::*CORE* plants (Fig. 1f), indicating that weak responses in younger plants are still *NbCORE*-dependent.

**Fig. 2:**
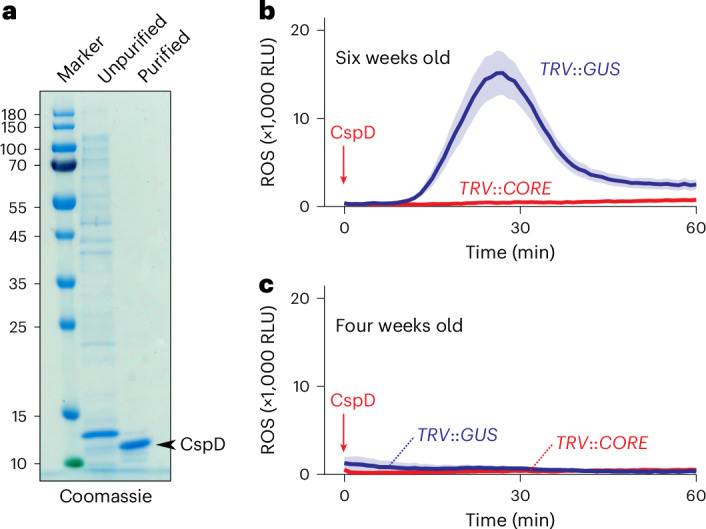
CspD triggers an *Nb*CORE-dependent oxidative burst in six-week-old *N. benthamiana.* **a**, Purification of CspD from *P. syringae* heterologously expressed in *E. coli.* *Pto*CspD was tagged with an N-terminal His–TEV tag, expressed in *E. coli* and purified by immobilized metal affinity chromatography. This experiment was repeated two times with similar results. **b**, CspD triggers an oxidative burst in six-week-old plants that is absent when *NbCORE* is silenced. **c**, CspD does not trigger an oxidative burst in four-week-old plants. In **b**,**c**, one-week-old *N. benthamiana* plants were infected with TRV carrying a fragment of *GUS* (*TRV*::*GUS*) or *NbCORE* (*TRV*::*CORE*). Leaf discs of four- and six-week-old plants were treated with 500 nM CspD, and ROS were measured in RLU. The lines represent the mean and the shading represents the s.e.m. of *n* = 6 leaf discs.

We next tested whether the purified CspD protein (Fig. 2a) could also trigger an *Nb*CORE-dependent oxidative burst. We expressed CspD with an N-terminal His tag in *E. coli* and purified this over Ni-NTA (Fig. 2a). Indeed, CspD used at similar concentrations as csp22 triggers an oxidative burst in six-week-old *TRV*::*GUS* plants, but not in *TRV*::*CORE* plants (Fig. 2b). The oxidative burst was nearly absent in four-week-old *TRV*::*CORE* and *TRV*::*GUS* plants (Fig. 2c), consistent with the low *Nb*CORE expression in younger plants. These data demonstrate that purified CspD triggers an *Nb*CORE-dependent oxidative burst, indicating that this sample does not contain other elicitors such as the bacterial flagellin of *E. coli*, which could also have triggered an oxidative burst in leaf discs of *N. benthamiana* because they express *Nb*FLS2.

### Apoplastic fluid quickly degrades CspD

**Fig. 3:**
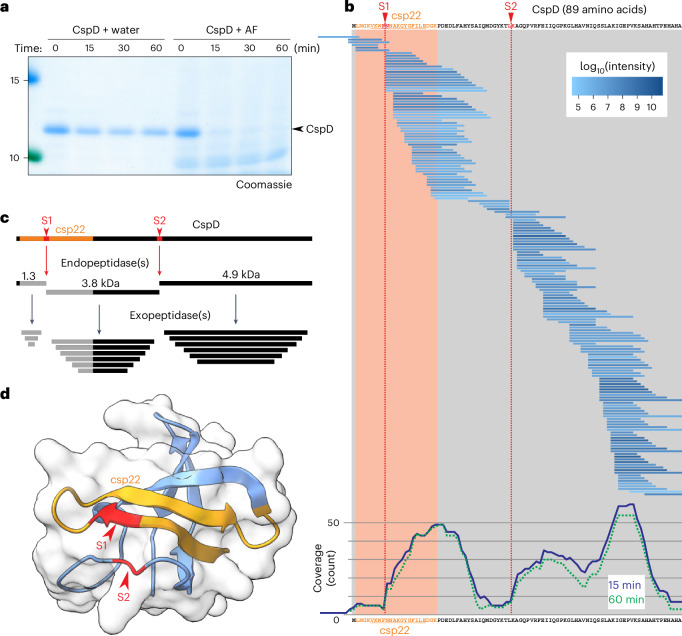
CspD is quickly degraded in AFs of *N. benthamiana.* **a**, Purified CspD is quickly degraded in AF of *N. benthamiana*. Purified CspD (2 μM) was incubated with water or AF for various times, and the products were analysed on a protein gel via Coomassie staining. This experiment was repeated four times, with similar results. **b**, Degradation products of CspD in AF detected by LC–MS/MS analysis. Purified CspD was incubated with AF isolated from *N. benthamiana* for 15 and 60 minutes. Proteins were precipitated with 80% acetone, and the peptide fraction (supernatant) was dried and analysed via LC–MS/MS. All CspD-derived peptides detected after 15 minutes of incubation were aligned with the CspD protein sequence. The red shaded region indicates csp22 of CspD. Likely initial cleavage sites are indicated by S1 and S2. The bottom graph shows the coverage of the CspD protein after incubation for 15 and 60 (dotted line) minutes, generated by counting the number of times that each residue was detected in the peptides. Peptide coverage graphs of 15 and 60 minutes’ incubation and the water controls are shown in Extended Data Fig. 2. **c**, Proposed degradation of CspD, first by endopeptidase(s) at sites S1 and S2 and then by exopeptidases. Cleavage at S1 inactivates the csp22 elicitor peptide. **d**, Positions of the S1 and S2 sites in the structural model of CspD. This structural model was obtained from the AlphaFold-predicted structure of CspD in the Uniprot database (entry Q87ZR9), trimmed for the cold-shock domain, with the csp22 peptide (orange) and S1 and S2 cleavage sites (red) as indicated. Source data

To test whether apoplastic fluids (AFs) could process CspD, we incubated purified CspD with AF isolated from four- to six-week-old *N. benthamiana* leaves and analysed proteins by SDS–PAGE. This experiment revealed a quick disappearance of intact CspD protein within 15 minutes (Fig. 3a), indicating that CspD is unstable in AF.

**Extended Data Fig. 2:**
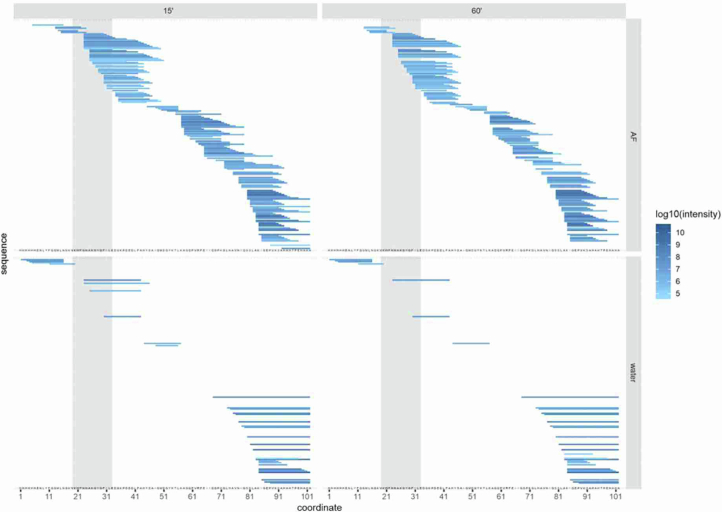
Detected CspD peptides. Coverages of detected CspD peptides after incubation of CspD in water (bottom) or AF (top) for 15 (left) and 60 (right) minutes.

To further investigate CspD degradation in AF, we analysed the released peptides via liquid chromatography–tandem mass spectrometry (LC–MS/MS) and mapped the CspD-derived peptides onto the CspD protein sequence. A total of 171 different CspD-derived peptides were detected, covering the entire CspD protein sequence (Fig. 3b). The peptides overlap and are staggered in three clusters, differing only in single residues removed from the N and carboxy termini (Fig. 3b and Extended Data Fig. 2). This peptide pattern indicates that CspD is cleaved at two sites (S1 and S2) by endopeptidases cleaving at VKWF ↓ NNAK and YKTL ↓ KAGQ, and that exopeptidases subsequently remove a series of single residues from either end (Fig. 3c). Cleavage at site S1 would cleave 10.0 kDa CspD into fragments of 1.3 and 8.7 kDa, whereas processing at S2 would result in 5.1 and 4.9 kDa fragments (Fig. 3c). Analysis of the CspD structural model^8^ indicates that site S1 is in the exposed β-sheet in middle of the csp22 elicitor sequence, and site S2 is in a nearby exposed loop (Fig. 3d).

### AF quickly inactivates csp22

**Fig. 4:**
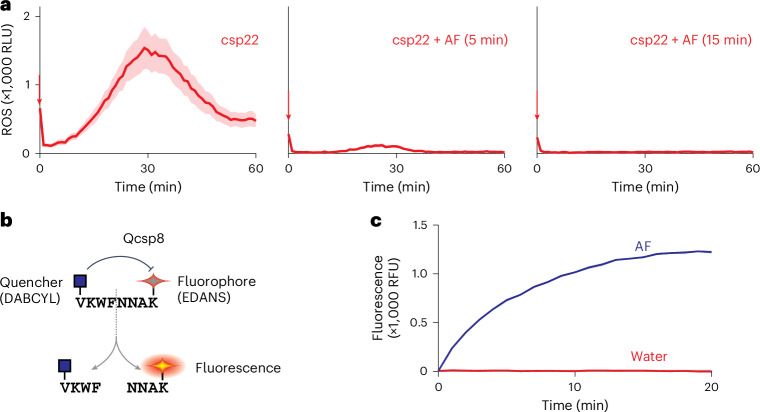
csp22 is quickly inactivated in AFs. **a**, Incubation in AF quickly inactivates the csp22 elicitor. AF was incubated with 500 nM csp22 peptide for 5 or 15 minutes and then added to leaf discs of six-week-old *N. benthamiana* plants floating on the luminol–HRP solution. Luminescence (RLU) was measured for 60 minutes with a plate reader. The lines represent the mean and the shading represents the s.e.m. of *n* = 12 replicates. **b**, Conceptual diagram of Qcsp8 showing the cleavage in csp22. **c**, Qcsp8 is quickly cleaved in AF isolated from *N. benthamiana* leaves. Qcsp8 (10 μM) was incubated in water or AF, and fluorescence was measured over time. The shading represents the s.e.m. of *n* = 3 replicates. RFU, relative fluorescence units.

Cleavage at site S1 of CSP within the csp22 epitope was unexpected and would inactivate the immunogenicity of csp22. To confirm csp22 cleavage, we incubated the csp22 peptide briefly in AF and then added this to leaf discs of csp22-responsive plants to detect remaining elicitor activity. Remarkably, elicitor activity drastically diminished within five minutes and was absent after 15 minutes of incubation (Fig. 4a), demonstrating that csp22 is unstable in AF.

To confirm cleavage in the middle of csp22, we obtained a custom-synthesized quenched octapeptide containing the eight residues surrounding the S1 site (VKWF ↓ NNAK) (Qcsp8). Cleavage of Qcsp8 would separate the N-terminal quencher (DABCYL) from the C-terminal fluorophore (EDANS), causing fluorescence (Fig. 4b). Incubation of Qcsp8 in AF causes a rapid increase of fluorescence compared with the water control (Fig. 4c), indicating that this octapeptide is cleaved in AF.

### Subtilases inactivate csp22, degrade CspD and cleave Qcsp8

**Fig. 5:**
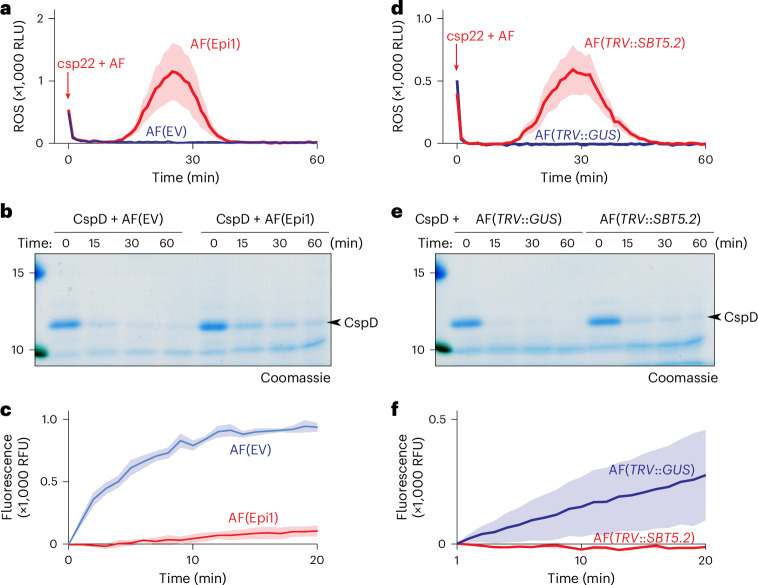
SBT5.2 subtilases are required for inactivating csp22, degrading CspD and cleaving Qcsp8. **a**, Inactivation of csp22 is suppressed in AF of plants overexpressing subtilase inhibitor Epi1. **b**, CspD degradation is reduced in AF of plants overexpressing subtilase inhibitor Epi1. This experiment was repeated once, with similar results. **c**, Cleavage of Qcsp8 is reduced in AF of plants overexpressing subtilase inhibitor Epi1. **d**, Inactivation of csp22 is suppressed in AF isolated from *TRV*::*SBT5.2* plants. **e**, CspD degradation is reduced in AF isolated from *TRV*::*SBT5.2* plants. **f**, Cleavage of Qcsp8 is reduced in AF isolated from *TRV*::*SBT5.2* plants. This experiment was repeated two times, with similar results. In **a**,**d**, AF was incubated with 500 nM csp22 peptide for 60 minutes and then added to leaf discs of six-week-old *N. benthamiana* plants floating on a luminol–HRP solution. Luminescence (RLU) was measured for 60 minutes with a plate reader. The lines represent the mean and the shading represents the s.e.m. of *n* = 6 (**a**) and *n* = 8 (**d**) replicates. In **b**,**e**, AF was incubated with 2 μM CspD protein for various time periods, separated on a 15% SDS–PAGE gel and stained with Coomassie. In **c**,**f**, AF was incubated with 10 µM Qcsp8 while fluorescence was measured with a plate reader. The lines represent the mean and the shading represents the s.e.m. of *n* = 4 samples. Source data

Being the largest class of proteases detected in the apoplast of *N. benthamiana*^17^, subtilases were tested for degrading csp22/CspD/Qcsp8 by taking advantage of subtilase inhibitor Epi1 of the oomycete potato blight pathogen *Phytophthora infestans*^18^. Elicitor activity was detected after incubation of csp22 peptide with AFs isolated from leaves transiently expressing Epi1 (AF(Epi1)), but not from leaves transiently transformed with the empty-vector control (AF(EV)) (Fig. 5a). This indicates that Epi1 blocks AF degradation of csp22, implying that apoplastic subtilases are responsible for degrading csp22.

We also tested whether CspD protein is more stable in the presence of Epi1 and found that CspD protein is stabilized in AF containing Epi1 and still degraded in AF of the EV control (Fig. 5b). Likewise, AF containing Epi1 was no longer able to cleave Qcsp8, unlike the AF(EV) control (Fig. 5c), indicating that subtilases are responsible for Qcsp8 cleavage. Taken together, these data indicate that subtilases are responsible for inactivating csp22, degrading CspD protein and cleaving Qcsp8.

### Subtilase SBT5.2 is required for degrading csp22 and CspD

As the most active subtilase in the apoplast of *N. benthamiana*^17–23^, subtilase SBT5.2 was further investigated for its role in csp22 degradation. We used VIGS to suppress *Nb*SBT5.2 expression in *N. benthamiana*^21,23^ and extracted AF from *TRV*::*SBT5.2* plants. *N. benthamiana* expresses three SBT5.2 homologues (a–c), which are all targeted with the 300-bp fragment of *SBT5.2a* present in *TRV*::*SBT5.2* (ref. ^23^). Elicitor activity of csp22 was detected upon incubation in AF from *TRV*::*SBT5.2* plants, but not in AF of *TRV*::*GUS* control plants (Fig. 5d), indicating that SBT5.2 is required for inactivating csp22. Likewise, CspD protein was more stable in AF of *TRV*::*SBT5.2* plants than in AF of *TRV*::*GUS* control plants (Fig. 5e), and the Qcsp8 peptide was no longer cleaved in AF of *TRV*::*SBT5.2* plants, unlike in the *TRV*::*GUS* control (Fig. 5f). Taken together, these data indicate that apoplastic subtilase SBT5.2 is required for inactivating csp22, degrading CspD protein and cleaving Qcsp8.

### Purified SBT5.2a inactivates csp22 and cleaves CspD and Qcsp8

**Fig. 6:**
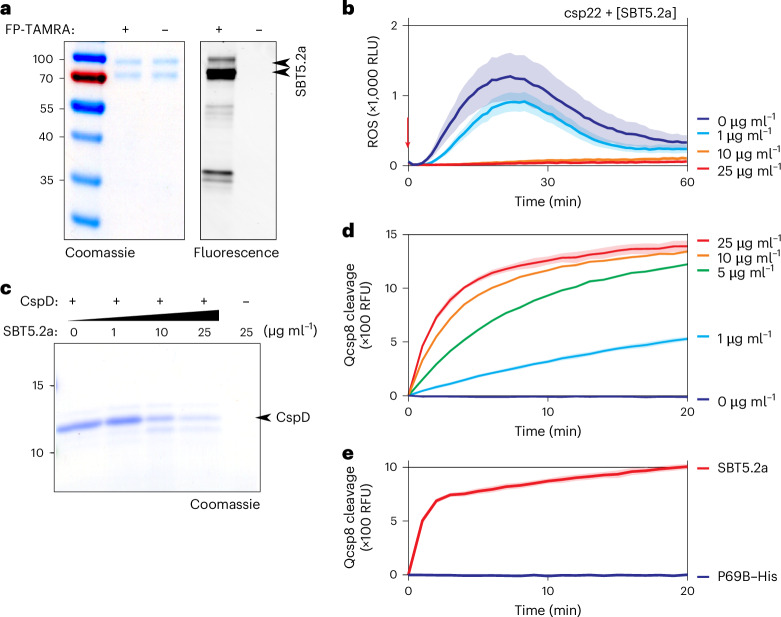
Purified SBT5.2a inactivates csp22 and cleaves CspD and Qcsp8. **a**, Purified SBT5.2a–His. His-tagged SBT5.2a was transiently expressed in *N. benthamiana* by agroinfiltration and isolated from AF at day 6 on Ni-NTA columns. Proteins were labelled with and without 0.2 μM FP-TAMRA for one hour and separated on SDS–PAGE stained with Coomassie or scanned for in-gel fluorescence. This experiment was repeated four times, with similar results. **b**, Purified SBT5.2a–His inactivates the csp22 elicitor. Purified SBT5.2a–His was incubated with 100 nM csp22 peptide for 45 minutes and then added to leaf discs of six-week-old *N. benthamiana* plants floating on a luminol–HRP solution. Luminescence (RLU) was measured for 60 minutes with a plate reader. The lines represent the mean and the shading represents the s.e.m. of *n* = 6 replicates. **c**, Purified SBT5.2a–His processes purified CspD protein. Purified CspD protein was incubated with various concentrations of purified SBT5.2a–His at room temperature for 40 minutes, separated on SDS–PAGE and stained with Coomassie. This experiment was repeated three times, with similar results. **d**, SBT5.2a cleaves Qcsp8. Various concentrations of purified SBT5.2a–His were incubated with 10 μM Qcsp8 while fluorescence was measured using a plate reader. The lines represent the mean and the shading represents the s.e.m. of *n* = 3 replicates. **e**, Qcsp8 is cleaved by SBT5.2a–His but not by P69B–His. 0.5 μg of purified SBT5.2a–His and P69B–His were incubated with 10 μM Qcsp8 while fluorescence was measured using a plate reader. The lines represent the mean and the shading represents the s.e.m. of *n* = 4 replicates. Source data

**Extended Data Fig. 3:**
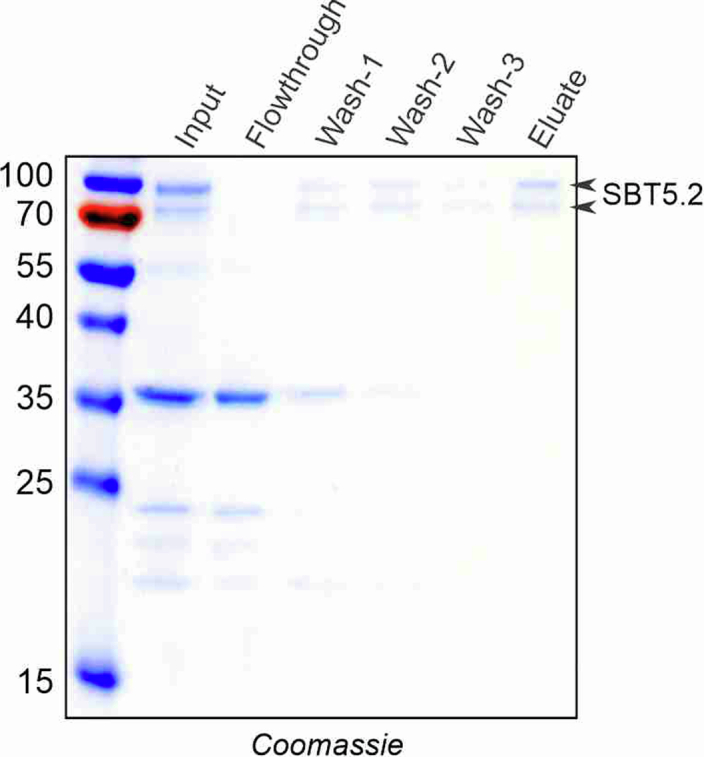
Purification of SBT5.2a-His from AF of agroinfiltrated plants. SBT5.2a-His was transiently expressed by agroinfiltration of *N. benthamiana*. AF was isolated at 6 dpi and purified over Ni-NTA. The column was washed with 50 mM imidazole and eluted with 250 mM imidazole. This experiment was repeated four times with similar results.

To determine whether SBT5.2 is also sufficient for inactivating csp22, degrading CspD protein and cleaving Qcsp8, we cloned the open reading frame encoding SBT5.2a (NbD038072 in the NbDE database^24^; NbL13g04590 in the LAB360 database^25^) fused to a C-terminal His tag into a binary vector and purified this protein from the AF of agroinfiltrated plants on Ni-NTA columns (Extended Data Fig. 3), resulting in purified SBT5.2a–His protein (Fig. 6a). We detected multiple isoforms that are labelled with the activity-based Ser hydrolase probe FP-TAMRA (Fig. 6a), indicating that they are derived from active proteases. Incubation of csp22 with different concentrations of purified SBT5.2a–His resulted in the dose-dependent inactivation of csp22 elicitor activity (Fig. 6b). Likewise, incubation of CspD with different concentrations of purified SBT5.2a–His resulted in the dose-dependent cleavage of CspD into a smaller isoform that was probably caused by cleavage at the S1 site because that cleavage would result in an 8.7 kDa product, whereas processing at S2 would generate two ~5 kDa products (Fig. 6b). Finally, incubation of Qcsp8 with different concentrations of purified SBT5.2a–His resulted in the dose-dependent cleavage of Qcsp8 (Fig. 6d). By contrast, Qcsp8 was not cleaved by purified P69B–His (Fig. 6e), a subtilase from tomato^21^, demonstrating the specificity of Qcsp8 cleavage by SBT5.2a. Taken together, these data indicate that apoplastic subtilase SBT5.2a can inactivate csp22, process CspD protein and cleave Qcsp8.

### Mutant *sbt5.2* plants have enhanced immune priming capacity

**Fig. 7:**
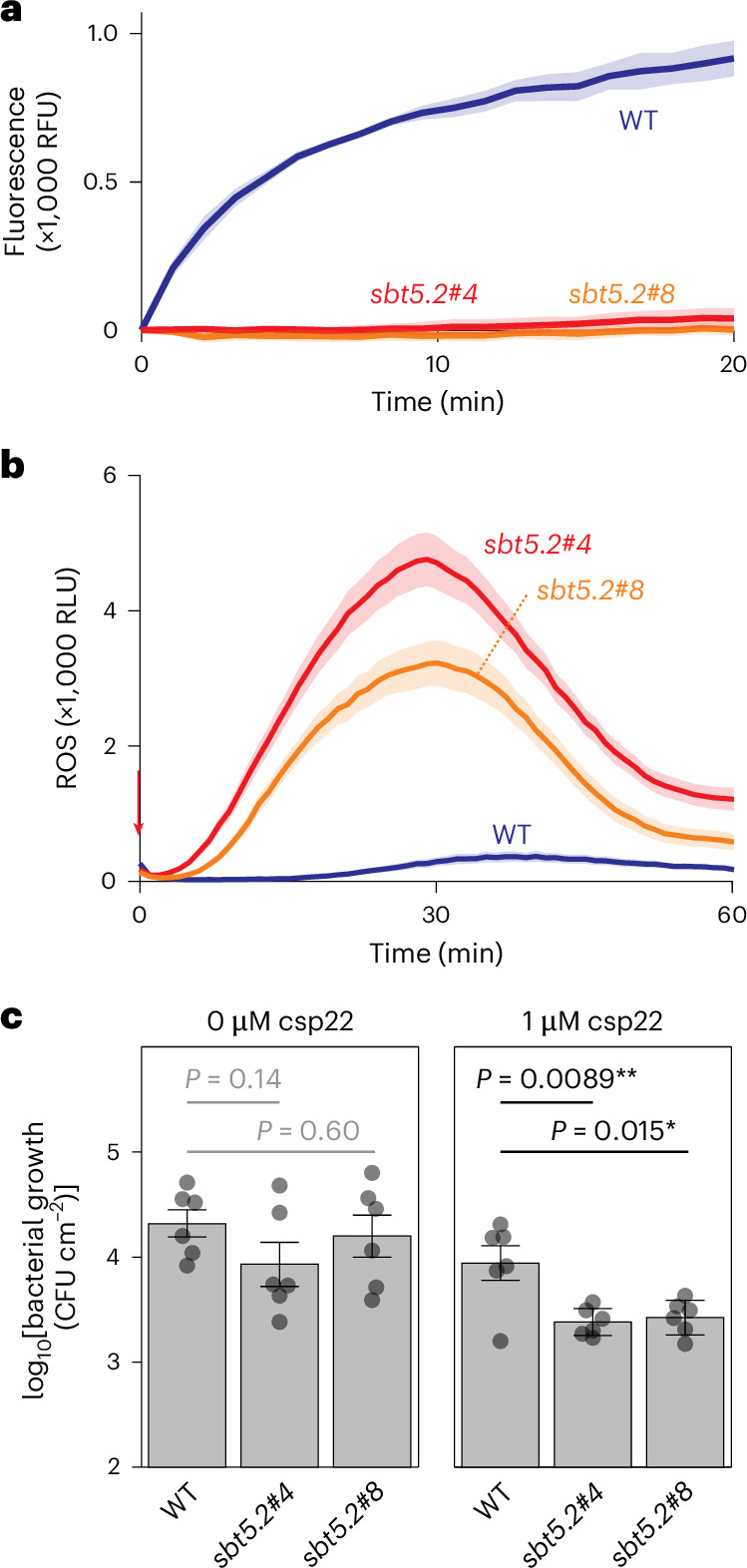
Increased stability of csp22 in *sbt5.2* triple mutants increases immunity. **a**, Qcsp8 is no longer cleaved in AF of *sbt5.2* mutants. Qcsp8 (10 µM) was incubated with AF of WT and *sbt5.2* mutant plants, and fluorescence was measured with a plate reader. The lines represent the mean and the shading represents the s.e.m. of *n* = 4 samples. **b**, Csp22 inactivation is reduced in AF isolated from both *sbt5.2* triple mutants. AFs of WT or *sbt5.2* mutant plants were incubated with 500 nM csp22 peptide for 30 minutes and then added to leaf discs of six-week-old *N. benthamiana* plants floating on luminol–HRP solution. Luminescence (RLU) was measured for 60 minutes with a plate reader. The lines represent the mean and the shading represents the s.e.m. of *n* = 12 replicates. **c**, Immunity induced by low csp22 concentration is increased in the *sbt5.2* mutant. Leaves of WT and *sbt5.2* mutant *N. benthamiana* were infiltrated with water or 1 μM csp22 and incubated for 24 hours and then injected with 1 × 10^5^ bacteria per ml of the flagellin mutant strain of *P. syringae* pv. *tomato* DC3000 (*Pto*DC3000Δ*fliC*Δ*hopQ*). Colony-forming units (CFU) were determined one day post infection. The error bars represent the s.e.m. of *n* = 6 replicates. The *P* values are from an unpaired, two-tailed Student’s *t*-test.

**Extended Data Fig. 4:**
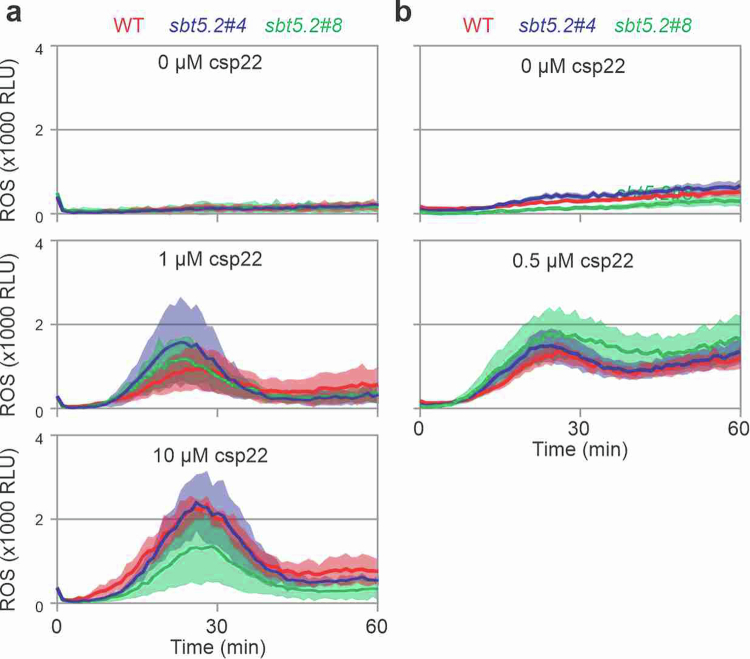
Mutant sbt5.2 plants still sense csp22. **a**, Leaf discs of 7-week old plants were treated with 0, 1 or 10 μM csp22 and luminescence with luminol was measured for one hour in relative light units (RLU). Error shades represent the STDEV of n = 4 replicates. **b**, Leaf discs of 7-week old plants were treated with 0, 1 or 10 μM csp22 and luminescence with LO-12 was measured for one hour in relative light units (RLU). Error shades represent the SE of n = 6 replicates.

**Extended Data Fig. 5:**
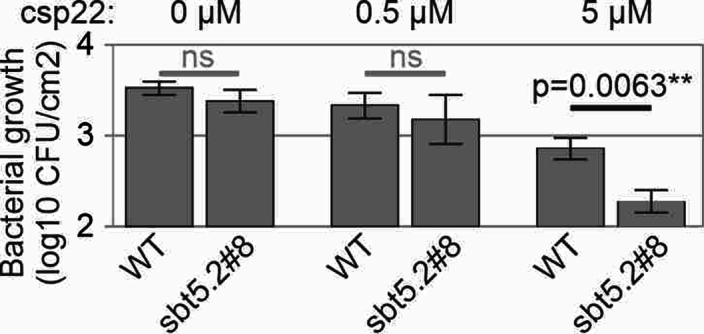
Immune priming is increased in the sbt5.2 mutant. Leaves of WT and *sbt5.2* mutant *N. benthamiana* were infiltrated with water or 0.5 or 5 μM csp22 and incubated for 24 hours and then injected with 1 × 10^5^ bacteria/mL of the flagellin mutant strain of *Pseudomonas syringae* pv. *tabaci* 6605 (*Pta*6605Δ*fliC*). Colony forming units (CFUs) were determined one day post infection (dpi). Error bars represent SE of n = 6 replicates.

To investigate the role of SBT5.2 subtilases in immunity, we took advantage of our recently introduced triple-knockout line lacking all three *SBT5.2* genes generated by genome editing^23^. AFs from these mutants are unable to cleave Qcsp8 (Fig. 7a) or inactivate csp22 (Fig. 7b). To assess the role of SBT5.2 in csp22-mediated immunity, we used the *fliC* mutant of *Pto*DC3000Δ*hopQ* (*Pto*DC3000Δ*hopQ*Δ*fliC*^26^) to avoid responses triggered by flagellin/FLS2 signalling. Infection assays, however, did not reveal altered bacterial growth in the *sbt5.2* mutant lines compared with wild-type (WT) plants (Fig. 7c). Elicitation with csp22 also did not reveal any changes in the oxidative burst (Extended Data Fig. 4a), even at low csp22 concentrations (Extended Data Fig. 4b). We then tested whether immune priming^27^ by csp22 is enhanced in *sbt5.2* mutants. Pretreatment with 1 µM csp22 followed by pathogen inoculation after 24 hours reduced bacterial growth in both *sbt5.2* mutant lines compared with WT plants (Fig. 7c). Likewise, pretreatment with 5 μM csp22 also reduced bacterial growth of the *fliC* mutant of *Pta*6605 (*Pta*6605Δ*fliC*^28^) more in *sbt5.2* mutant plants than in WT plants (Extended Data Fig. 5). These data demonstrate that the degradation of csp22 by SBT5.2 in WT plants increases plant susceptibility to bacterial infection.

### Polymorphisms in csp22 dictate SBT5.2 sensitivity

**Fig. 8:**
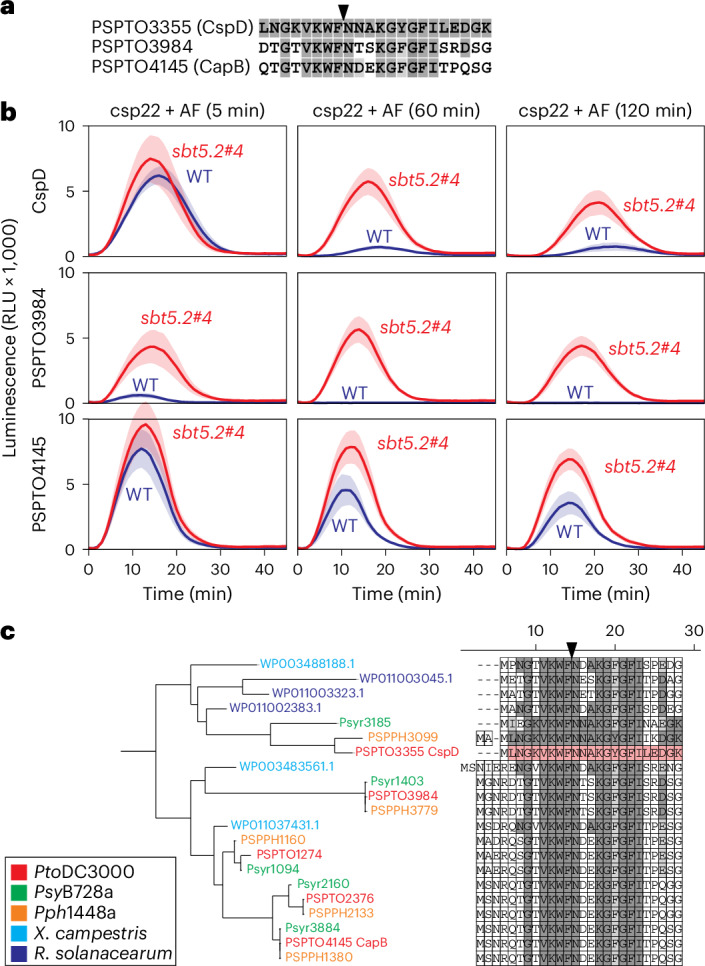
Differential processing of csp22 peptides by SBT5.2. **a**, Alignment of csp22 sequences from CspD (PSPTO03355), PSPTO3984 and PSPTO4145 from *Pto*DC3000. **b**, The csp22 peptides (1,000 nM) were incubated with AF from WT plants and the *sbt5.2#4* mutant for 5, 60 and 120 min, diluted tenfold and then added to leaf discs of *N. benthamiana* floating in HRP and luminol. ROS burst was measured immediately with a plate reader over 45 minutes. The lines represent the mean and the shading represents the s.e.m. of *n* = 8 replicates. **c**, Phylogeny of CSPs with an alignment of enclosed csp22 sequences of *Pto*DC3000 (red), *P. syringae* pv. *syringae* B728a (*Psy*B728a; green) and *P. syringae* pv. *phaseolicola* 1448A (*Pph*1448a; orange), as well as *Xanthomonas campestris* ATCC33913 (cyan) and *Ralstonia solanacearum* GMI1000 (blue). The highlighted residues are identical (dark grey) or similar (light grey) to the csp22 sequence of CspD. In **a**,**c**, arrowhead indicates putative cleavage site.

**Extended Data Fig. 6:**
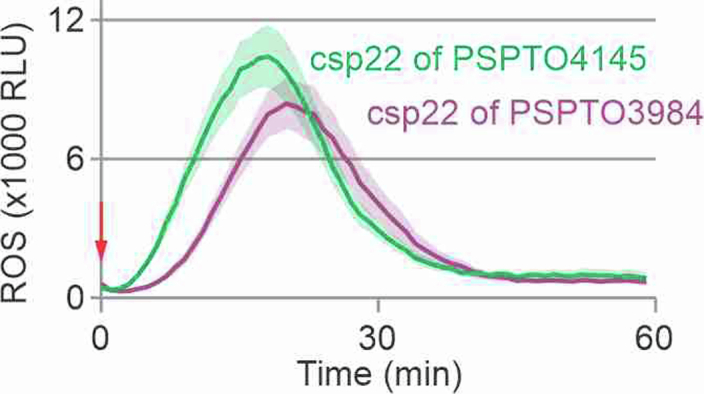
Also csp22 peptides from other CSPs trigger an oxidative burst. Leaf discs of 6-week old plants were treated with 500 nM csp22 of PSPTO3984 or PSPTO4145, and ROS was measured in relative light units (RLU). Error shades represent the SE of n = 6 replicates.

To investigate whether other csp22 elicitor peptides present in CSPs of *Pto*DC3000 have differential sensitivity to SBT5.2-mediated inactivation, we tested two more csp22 peptides that are distinct from csp22 of CspD: csp22 of CapB (PSPTO4145) and PSPTO3984, which differ by 11 and 10 residues from csp22 of CspD, respectively (Fig. 8a). Both csp22 peptides trigger ROS bursts in leaf discs of six-week-old *N. benthamiana* plants (Extended Data Fig. 6). All three peptides are inactivated when incubated in AF, but the speed of inactivation differs per peptide (Fig. 8b). When incubated with AF of WT plants, the csp22 peptide of PSPTO3984 is more quickly degraded than that of CspD, whereas the degradation of csp22 of CapB is slower than that of CspD (Fig. 8b). However, all peptides are stabilized to similar levels when incubated in AF of the *sbt5.2* mutant (Fig. 8b), indicating that SBT5.2 subtilases contribute to the degradation of all three csp22 peptides. Because the residues preceding the cleavage site are identical between CspD, PSPTO3984 and CapB, the reduced processing of CapB by SBT5.2 is probably caused by the presence of acidic residues (Asp16 and Glu17) after the cleavage site. These data indicate that the polymorphism in csp22 peptides can lead to varying sensitivity to SBT5.2-mediated cleavage, resulting in different stabilities in the plant apoplast. The alignment with csp22 peptides from different bacterial plant pathogens indicates that the variation in csp22 stabilities might be similar between different bacterial plant pathogens (Fig. 8c).

## Discussion

This work has demonstrated that immunogenic csp22 peptides are quickly inactivated by subtilase SBT5.2 in the apoplast. This discovery indicates that SBT5.2 dampens PTI by degrading csp22 and that bacteria may take advantage of SBT5.2 degrading elicitors to evade recognition.

**Extended Data Fig. 7:**
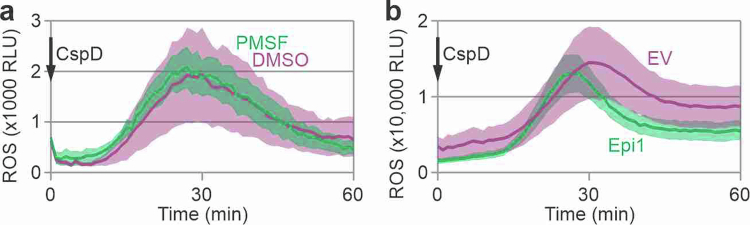
Subtilase inhibitors do not block CspD perception. **a**, Leaf discs of *N. benthamiana* were treated with 0.5 mM PMSF or 1% DMSO when floating on luminol-HRP solution and were triggered with 500 nM CspD protein and the release of reactive oxygen species (ROS) was detected by luminescence. Error shades represent SE of n = 6 replicates. **b**, Leaf discs taken from agroinfiltrated leaves expressing Epi1 or empty vector (EV) control, taken at 3 dpi, floating on luminol-HRP solution, were triggered with 500 nM CspD protein and the release of reactive oxygen species (ROS) was detected by luminescence. Error shades represent SE of n = 6 replicates.

This project was initiated to investigate whether peptide elicitors require proteolytic processing for their release from their precursors. We anticipated this since most peptide elicitors are not accessible within the folded precursor^8^, and many peptide hormones have to be released from precursors to be perceived elsewhere by receptors^29,30^. The CSP, however, is very small, and much of the csp22 peptide is already exposed (Fig. 3d). In addition, since the csp22 peptide is at the N terminus, only C-terminal processing would be required for csp22 release. However, the timing of the oxidative burst upon CspD is not significantly different from that of csp22 (Figs. 1c,e and 2b), and we were unable to block CspD perception with subtilase inhibitors PMSF or Epi1 (Extended Data Fig. 7). And although csp22 binding to CORE has been demonstrated^11^, unprocessed CSPs are likely to bind to CORE, similar to how flagellin protein can bind FLS2 (ref. ^31^). The hypothesis that processing is not required for CSP perception is also consistent with the distribution of detected peptides over the CspD protein when incubated with AF as the detected peptide pattern suggests that there were only two initial processing sites (S1 and S2; Fig. 3b). Whereas processing at site S1 inactivates csp22, site S2 is far beyond the csp22 peptide. Furthermore, incubation of purified CspD with purified SBT5.2a indicates that site S1 is cleaved first and cleavage at S2 might be a secondary cleavage event (Fig. 6c). These observations indicate that processing might not be important for the release of the csp22 elicitor. By contrast, the inactivation of the csp22 epitope by SBT5.2 at site S1 seemed a more relevant process.

Indeed, subsequent experiments with csp22 confirmed that csp22 is unstable in the apoplast. We provided multiple lines of evidence that SBT5.2s are both required and sufficient for inactivating csp22. Inactivation of csp22 in AF can be blocked with SBT inhibitor Epi1, by silencing *SBT5.2*s or by inactivating *SBT5.2*s by genome editing. Conversely, purified SBT5.2a is sufficient to inactivate csp22 and can cleave the quenched octapeptide carrying the presumed S1 cleavage site. Also, the processing of CspD protein by purified SBT5.2a suggests that the S1 site is cleaved by SBT5.2. The S1 site in csp22 (VKWF ↓ NNAK) is consistent with the known promiscuity of this protease. SBT5.2 is also required for cleaving the prodomain of proRcr3 in the region carrying the sequence (EFKI-)NDLSDDYM(-PSN) irrespective of random mutagenesis in this region^21^, and purified SBT5.2a was found to cleave various recombinant proteins at sites TTLF ↓ GVPI, YNYY ↓ DFYD, YNYH ↓ YMDV, YHYM ↓ DVWG, TTVT ↓ VSSA, VTVS ↓ SAST, MFLE ↓ AIPM and AIPM ↓ SIPP^20^. Although there are often hydrophobic residues at positions P4, P2 and P1 (Val, Trp and Phe in csp22, respectively), there are several exceptions, indicating that SBT5.2 might be promiscuous and that it is rather the combination of residues in a peptide that may dictate SBT5.2 cleavability. The promiscuity of SBT5.2 is consistent with observations made for *Arabidopsis* SBT5.2, which was found to process the precursors of Inflorescence Deficient in Abscission (proIDA^32^) and Epidermal Patterning Factor (proEPF2; ref. ^33^), as well as flagellin^34^, at sites that do not share much homology.

The consequence of csp22 processing is that the csp22 levels are suppressed by SBT5.2 and that, consequently, the immune response is dampened by SBT5.2 s. Indeed, priming by low csp22 concentrations induces PTI only in *sbt5.2* mutants (Fig. 7c), confirming that SBT5.2 dampens csp22-triggered PTI. A dampened immune response may benefit the plant by restricting costly immune responses to the site of pathogen infection by avoiding the diffusion of peptide elicitors. Likewise, csp22 degradation will also result in a more temporal immune response, so that PTI is no longer triggered in plants after an infection has cleared. Both temporal and spatial dampening of immune response would avoid unnecessary activation of costly immune responses and make more resources available to promote plant growth.

But the fact that SBT5.2s inactivate csp22 is also a clear benefit to the pathogen, and this implies that pathogens might take advantage of plant SBT5.2 to degrade their elicitors. The relevance of elicitor degradation is testified by the fact that *P. syringae* secretes AprA, a metalloprotease that inactivates flg22, the main elicitor from flagellin^35^. Selection for destabilization also occurred in Avr4, a protein secreted by the fungal tomato pathogen *Cladosporium fulvum* that is recognized by the Cf-4 immune receptor in tomato. Immune evasion resulted in virulent races that predominantly carry substitutions of Cys residues that destabilize Avr4 in the apoplast but maintain its ability to protect chitin against chitinases^36,37^. We were also able to demonstrate that SBT5.2 cleaves the flg22 epitope in flagellin, inactivating its immunogenicity^38^. Interestingly, the fact that both CSP and flagellin are cleaved first in the immunogenic epitopes instead of elsewhere is remarkable and might have resulted from evolutionary pressure to avoid recognition.

Interestingly, we detected some variation in the stability of the csp22 peptides, although all peptides are eventually degraded by SBT5.2. Since the tested peptides all carry the VKWF tetrapeptide before the cleavage site, the differential stability is probably caused by variation in residues after the cleavage site. This indicates that the NDEK tetrapeptide after the cleavage site reduces SBT5.2 sensitivity, whereas the NTSK sequence increases SBT5.2 sensitivity when compared with the NNAK tetrapeptide from CspD. These observations indicate that acidic residues (Asp or Glu) at the P2′ and P3′ positions may reduce the SBT5.2 sensitivity of csp22 peptides. The different stabilities of the csp22 peptides implies that the relative concentrations of these different csp22 peptides will change after infection and at a distance from the infection site.

A recent pangenomic study on the diversity of csp22 in bacteria revealed that csp22 exhibits significant copy and epitope variation^39^. Interestingly, 25% of the tested csp22 peptides were not immunogenic in tomato carrying the *CORE* receptor, and some of these peptides were found to suppress csp22-mediated PTI by blocking the perception of immunogenic csp22. Remarkably, these non-immunogenic csp22 peptides have even more variation surrounding the S1 site, indicating that antagonistic csp22 peptides might have an enhanced stability to increase their ability to interfere with PTI signalling. These predictions, however, are all based on assays with synthetic csp22 peptides. Although five *CSP* genes are expressed in *Pto*DC3000 during infection, and derived peptides have been detected in crude AFs from infected plants by proteomics^39^, it remains to be shown which CSPs are perceived during infection. Experiments to delete *CSP* genes or alter the csp22 sequence are challenging because CSPs are collectively essential for bacterial survival^40,41^, and the RNA-binding motif overlaps with the csp22 epitope.

SBT5.2 is the most active subtilase in the apoplast of *N. benthamiana*, and its role in degrading csp22 epitopes (this work) and flg22 epitopes^38^ has now been demonstrated. Without preincubation in AF, we did not detect an altered ROS burst upon csp22/flg22 signalling in the *sbt5.2* mutant, indicating that SBT5.2 is not involved in signalling itself. We did also not detect any macroscopic developmental phenotype of *sbt5.2* mutants^23^. *Arabidopsis* *sbt5.2* mutants also grow normally^32^. Given its abundance and promiscuity, we hypothesize that SBT5.2 might mediate the removal of unstable proteins in the apoplast to maintain extracellular protein homeostasis. The identification of proteins accumulating in the apoplast of *sbt5.2* mutant plants may therefore provide more insights into the endogenous role of SBT5.2.

The fact that a single bacterium produces multiple CSPs including csp22 peptides that have different stability and immunogenicity indicates that the outcome of interactions involving CSPs is complicated. These interactions will be further complicated by the variation in apoplastic proteases between plant species and upon the secretion of immune proteases. These interactions can be further fine-tuned by the secretion of protease inhibitors by pathogens. Although the csp22 sequences are evolutionarily constrained by the fact that they contain the RNA-binding motif required for the intrinsic function of CSP^9^, these observations indicate that csp22 variation might underlie a fascinating natural battlefield that is pivotal for the outcome of plant–bacterium interactions.

## Methods

### Plants

*Nicotiana benthamiana* plants (LAB genotype) were grown at 21 °C (night) and 22–23 °C (day) under a 16 h light (80–120 μmol m^−2^ s^−1^)/8 h dark routine in a greenhouse until use.

### Molecular cloning

All the primers and plasmids used are summarized in Supplementary Tables 1 and 2, respectively. Expression vector pJK155 was generated by cloning inserts of pJP001, pJK120, pFGH029 and pJP002 into pJK082 using a BsaI Golden Gate reaction, resulting in pJK155 (pET28b–T7::OmpA–His–TEV–EPIC1). Open reading frames of *CapB* and *CspD* from *Pto*DC3000 were amplified from genomic DNA of *Pto*DC3000 using the primers listed in Supplementary Table 1 and cloned into pJK155, which was linearized by PCR with primers 5′-gcttggatccggctgctaac-3′ and 5′-accttggaagtataggttttcgtg-3′ using the Gibson ligation method, resulting in pCC03 (pET28b–T7::OmpA–His–TEV–CapB) and pCC04 (pET28b–T7::OmpA–His–TEV–CspD). The open reading frame of *Nb*SBT5.2a was commercially synthesized with a C-terminal 6HIS tag (Twist Bioscience; Supplementary Table 1) and assembled with the Golden Gate–compatible vector pJK001c binary vector^21^, the 35S promoter module (pICH51288) and the 35S terminator module (pICH41414) in a BsaI reaction resulting in expression plasmid pPB097 for transient protein expression of SBT5.2a–His in *N. benthamiana*. This plasmid was transformed into *E. coli* DH10β for amplification, purified and then transformed into *Agrobacterium tumefaciens* GV3101(pMP90). Transformants were selected on plates of LB-agar medium containing 25 µg ml^−1^ rifampicin, 10 µg ml^−1^ gentamycin and 50 µg ml^−1^ kanamycin.

### Expression and purification of CspD

Plasmid pCC04 was transformed into the Rosetta strain of *E. coli*, protein expression was induced with 0.4 mM IPTG at 20 °C and proteins were purified on HisPur Ni-NTA Resin (Thermo Scientific) according to the manufacturer’s instructions. Protein purity was verified by protein gel electrophoresis followed by Coomassie staining and western blotting using anti-His (HRP) antibody (Miltenyi Biotec). Signals were generated by chemiluminescence using Clarity ECL Western Blotting Substrate (BioRad) and detected with the ImageQuant LAS 4000 (GE Healthcare). Purified proteins were further concentrated using an Amicon Ultra centrifugal filter device (3 kDa MW cut-off, Millipore). Protein quantity was measured using Bradford method (Sigma-Aldrich). Proteins were stored in aliquots at −80 °C until use.

### Transient protein expression in *N. benthamiana*

To express proteins transiently in *N. benthamiana* by agroinfiltration, overnight cultures of *A. tumefaciens* GV3101(pMP90) carrying binary plasmids to express Epi1 (pFGH048; ref. ^42^) or SBT5.2a–HIS (pPB097) were harvested by centrifugation. Cells were resuspended in induction buffer (10 mM MgCl_2_, 10 mM MES pH 5.0 and 150 µM acetosyringone) and mixed (1:1) with agrobacteria carrying the silencing inhibitor P19 at OD_600_ = 0.5. After 1 h at 21 °C, the cells were infiltrated with a needleless syringe into the abaxial side of three leaves of four-week-old *N. benthamiana*. The leaves were harvested and processed at the indicated days after agroinfiltration.

### Isolation of AFs

AFs were collected as described previously^43^. Leaves from WT, agroinfiltrated or VIGS-silenced plants were submerged in ice-cold water and infiltrated by applying a vacuum for 5 min. The surface of water-infiltrated leaves was dried with absorbing paper, and the leaves were carefully mounted in an empty 20 ml syringe and placed in a 50 ml tube. AFs were collected by centrifugation at 2,000 *g* at 4 °C for 20 min and used immediately or flash-frozen and stored at −20 °C.

### SBT5.2a–His and P69B–His purification

Four-week-old *N. benthamiana* leaves were infiltrated with a 1:1 mixture (final OD_600_ = 0.5 for each) of *A. tumefaciens* GV3101 containing the silencing suppressor P19 and pPB097 or pJP008. AF containing SBT5.2a–His or P69B–His was extracted six days after infiltration and purified as previously described^44,45^.

### ROS assays

The ROS burst assay was performed as previously described^43^ except that L-012 (Wako Chemical) was used instead of luminol and the diameter of leaf discs used here was 4 mm rather than 6 mm. Briefly, after incubation in water overnight, one leaf disc (4 mm in diameter) was added to 100 µl of solution containing 25 ng µl^−1^ L-012, 25 ng µl^−1^ HRP and specified elicitor treatments. For assays with elicitors treated with AFs, 500 nM csp22 or purified CspD was incubated in AFs from the specified *N. benthamiana* leaves (WT, agroinfiltrated or VIGS-silenced) for one hour at room temperature with slight shaking. After incubation, 25 ng µl^−1^ L-012 and 25 ng µl^−1^ HRP were added to the AFs. Chemiluminescence was measured immediately with an Infinite M200 plate reader (Tecan) every minute for one hour.

### Peptide synthesis

All peptides used were custom-synthesized by GenScript and are summarized in Supplementary Table 3.

### Qcsp8 assays

Qcsp8 (VKWFNNAK) from CspD was commercially synthesized with a DABCYL N-terminal modification and an EDANS C-terminal modification (GenScript) at a purity of 95.7%. It was resuspended in DMSO at a concentration of 1 mM. This stock solution was further diluted in water to a concentration of 200 µM. AFs or purified SBT5.2a or P69B were mixed with Qcsp8 at a final concentration of 10 µM in a volume of 100 µl, and fluorescence was measured immediately or after the indicated incubation time at 21 °C using an Infinite M200 plate reader (Tecan) with an excitation wavelength of 335 nm and an emission wavelength of 493 nm.

### In vitro degradation assays of CspD by AFs

Purified CspD (stock concentration, 10 μM; final concentration, 2 μM) was incubated in AFs from the specified *N. benthamiana* leaves (WT, agroinfiltrated or VIGS-silenced) for the indicated times at room temperature with slight shaking. Proteins were analysed via SDS–PAGE and Coomassie staining.

### Peptide release from digestion of CspD in AF

First, 10 ng µl^−1^ of purified CspD protein produced in *E. coli* was incubated in AF from WT *N. benthamiana* for 15 min or 60 min at room temperature. CspD and AF alone were used as negative controls, and two technical replicates were included for each treatment or control. After incubation, samples for the analysis of endogenously digested peptides in the AF were generated by supplementing the AF with four volumes of MS-grade acetone, followed by incubation on ice for one hour and centrifugation at 18,000 *g* for 15 min. Four fifths of the supernatants were then transferred to fresh Eppendorf tubes, and the acetone was evaporated by vacuum centrifugation. The dried peptide samples were then dissolved in 0.1% formic acid and immediately analysed without further clean-up. LC–MS/MS analysis and peptide identification were performed as previously described^43^ except that MS/MS spectra data were searched against the sequence of CspD protein. Identified CspD peptides in samples of CspD incubated in AF were normalized by subtraction of the data generated from negative controls and aligned with the CspD protein sequence.

### LC–MS/MS

Each sample was analysed on an Orbitrap Elite instrument (Thermo)^46^ that was coupled to an EASY-nLC 1000 LC system (Thermo) and an Orbitrap Fusion Lumos (Thermo) coupled to an EASY-nLC 1200 LC system (Thermo). The LC systems were operated in the one-column mode. The analytical column was a fused silica capillary (75 µm × 46 cm) with an integrated fritted emitter (15 µm; CoAnn Technologies) packed in-house with Kinetex C18-XB core shell 1.7 µm resin (Phenomenex). The analytical column was encased by a column oven (Sonation) and attached to a nanospray flex ion source (Thermo). The column oven temperature was adjusted to 50 °C during data acquisition. The LC system was equipped with two mobile phases: solvent A (0.2% formic acid in water) and solvent B (0.2% formic acid, 19.8% water and 80% acetonitrile). All solvents were of UPLC grade (Honeywell). Peptides were directly loaded onto the analytical column with a maximum flow rate that would not exceed the set pressure limit of 980 bar (usually around 0.6–1.0 µl min^−1^). Peptides were subsequently separated on the analytical column by running a gradient of solvent A and solvent B.

### Peptide and protein identification using MaxQuant

RAW spectra were submitted to an Andromeda^47^ search in MaxQuant (v.2.0.2.0) using the default settings^48^. Label-free quantification and match-between-runs were activated^49^. The MS/MS spectra data were searched against a project-specific database containing two sequences of interest (ACE_0686_SOI_v01.fasta; two entries). All searches included a contaminants database search (as implemented in MaxQuant, 245 entries). The contaminants database contains known MS contaminants and was included to estimate the level of contamination. Andromeda searches allowed oxidation of methionine residues (16 Da) and acetylation of the protein N terminus (42 Da). No dynamic modifications were selected. Enzyme specificity was set to ‘unspecific’. The instrument type in Andromeda searches was set to Orbitrap, and the precursor mass tolerance was set to ±20 ppm (first search) and ±4.5 ppm (main search). The MS/MS match tolerance was set to ±0.5 Da. The peptide spectrum match false discovery rate and the protein false discovery rate were set to 0.01 (on the basis of the target-decoy approach). The minimum peptide length was 6 amino acids, and the maximum length was 36. For protein quantification, unique and razor peptides were allowed. Modified peptides were allowed for quantification. The minimum score for modified peptides was 40. Label-free protein quantification was switched on, and unique and razor peptides were considered for quantification with a minimum ratio count of 2. Retention times were recalibrated on the basis of the built-in nonlinear time-rescaling algorithm. MS/MS identifications were transferred between LC–MS/MS runs with the ‘match between runs’ option, in which the maximal match time window was set to 0.7 min and the alignment time window set to 20 min. The quantification is based on the ‘value at maximum’ of the extracted ion current. At least two quantitation events were required for a quantifiable protein. Further analysis and filtering of the results were done in Perseus v.1.6.10.0 (ref. ^50^). Comparison of protein group quantities (relative quantification) between different MS runs is based solely on the label-free quantifications as calculated by MaxQuant with the MaxLFQ algorithm^49^.

### VIGS

*N. benthamiana* plants silenced for *NbCORE*, *SBT5.2*, *GUS* (negative control) and phytoene desaturase (positive control) were generated using VIGS as previously described^13,23^. Briefly, overnight cultures of *A. tumefaciens* GV3101 were collected and resuspended in agroinfiltration buffer (10 mM MgCl_2_, 10 mM MES pH 5.0 and 100 mM acetosyringone). Suspensions of bacteria containing *TRV2gg*::*NbCORE*, *TRV2*::*SBT5.2* and *TRV2*::*GUS* were mixed 1:1 separately with bacteria containing *TRV1* at OD_600_ = 0.5 for each bacterium. After incubation for one hour at room temperature, the mixed cultures were infiltrated into true leaves of two-week-old *N. benthamiana* plants. The infiltrated seedlings were grown in a growth chamber until use.

### Labelling of active subtilases

FP-TAMRA (Thermo Scientific) was prepared as 10 µM stock solutions in dimethyl sulfoxide. Labelling was performed as described previously^43^. For fluorescence gel imaging, the AFs were incubated with 0.2 µM probes for 1 h at room temperature in the dark. The labelling reactions were stopped by adding 4× gel loading buffer (200 mM Tris-HCl (pH 6.8), 400 mM DTT, 8% SDS, 0.4% bromophenol blue and 40% glycerol) and heating at 90 °C for 5 min. The proteins were separated on SDS–PAGE, and fluorescence was detected from protein gels using the Typhoon FLA 9000 scanner (GE Healthcare Life Sciences) using Cy3 settings (532 nm excitation and 610PB filter).

### Infection assays

For the infection assays, csp22 peptides were diluted in water. Three fully expanded leaves of three- to four-week-old *N. benthamiana* plants were infiltrated with different concentrations of csp22 peptide or with water as a mock control. 24 h later, infiltrated leaves were infiltrated with 10^5^ CFU ml^−1^ *Pto*DC3000(ΔhQ) or *Pta*6605. The next day, three leaf discs were punched with a cork borer from each infected leaf and surface-sterilized with 15% hydrogen peroxide for 2 min. The leaf discs were then washed twice in MilliQ and dried under sterile conditions. The leaf discs were placed into a 1.5 ml safe-lock Eppendorf tube with three 3-mm-diameter metal beads and 1 ml of MilliQ. The tubes were placed in tissue lyser for 5 min at 30 Hertz per second. 20 µl of undiluted tissue and serial dilutions were plated on LB-agar plates containing Pseudomonas CFC Agar Supplement (Oxoid SR0103). The plates were allowed to dry and incubated at 28 °C for two days, and then colonies were counted. *P* values were calculated using two-tailed Student’s *t*-tests to compare bacterial growth between leaves from WT and *sbt5.2* mutant plants.

### Statistics

All values shown are mean values, and the error intervals shown represent the standard error of the mean, unless otherwise indicated. All experiments have been reproduced, and representative datasets are shown.

### Phylogenetic analysis

Proteomes of *P. syringae* strains were obtained from the pseudomonas.com database^51^, and proteomes of *Ralstonia solanacearum* and *Xanthomonas campestris* were obtained from the RefSeq database^52^. Cold-shock-domain-containing proteins were identified using the hmmsearch function from HMMER v.3.3.2 (ref. ^53^) with the PF00313 (cold-shock DNA-binding domain) Pfam profile^54^. Only sequences with a recognizable csp22 epitope were retained. Protein sequences were aligned using MAFFT v.7 with the L-INS-i algorithm^55^. A maximum likelihood phylogenetic tree was constructed using IQ-TREE v.2 (ref. ^56^) with the best-fit substitution model from ModelFinder^57^. Branch support values were calculated using ultrafast bootstrap with 1,000 replications. Phylogenetic trees were visualized using iTOL^58^ with midpoint rooting.

### Reporting summary

Further information on research design is available in the Nature Portfolio Reporting Summary linked to this article.

## Supplementary information

Supplementary Information Supplementary Tables 1–3.

Reporting Summary

## Source data

Source Data Fig. 3a Uncropped gel for Fig. 3a.

Source Data Fig. 5b,e Uncropped gel for Fig. 5b,e.

Source Data Fig. 6a,c Uncropped gel for Fig. 6a,c.

## Data Availability

The MS proteomics data for the on-bead digestions have been deposited to the ProteomeXchange Consortium via the PRIDE^59^ partner repository (https://www.ebi.ac.uk/pride/archive/) with the dataset identifier PXD048912. Source data are provided with this paper.
