# Supplementary material for: Extracellular plant subtilases dampen cold-shock peptide elicitor levels
Source: Nat Plants. 2024 Oct 11;10(11):1749–60. doi: 10.1038/s41477-024-01815-8 (PMC11570497; doi:10.1038/s41477-024-01815-8)
Supplement: Supplementary file 1 — Supplementary Tables 1–3. [file 41477_2024_1815_MOESM1_ESM.pdf]

nature plants

Supplementary information https://doi.org/10.1038/s41477-024-01815-8

Extracellular plant subtilases dampen cold-shock peptide elicitor levels

In the format provided by the authors and unedited

Table S1 Used synthetic oligonucleotides

**Name**	**Sequence (5'-3')**
CapB_F	CTATACTTCCAAGGTATGTCCAATCGCCAAACTGG
CapB_R	CAGCCGGATCCAAGCTTAGATAACTTGAACTTCTTCAGCT
CspD_F	CTATACTTCCAAGGTATGCTTAACGGAAAAGTCAAGTGGT
CspD_R	CAGCCGGATCCAAGCTCAAGCGTGTGCGTGTTCA
pJK155_linearR1	ACCTTGGAAGTATAGGTTTTCGTG
pJK155_linearF1	GCTTGGATCCGGCTGCTAAC
SBT5.2a-His	ATGAAAGGAATTATATCTTTGTTTTTCTGCTTTTCTCTTTTCATTGTCTCTTTCATATTAAGAG<br>AAGCCGACTCAGCTTCTCAAGCACAAAACAATGGAATTTATATTGTTTATATGGGTGCTGCA<br>GCTTCATCTAATGGTGGTACCAGACATGATCAAGCGCGGCTTATCAGCTCCTTGATCAGAAG<br>GAACAAGCATGCAGTGGTACACAGCTACAACAATGGTTTCTCAGGATTCGCGGCACGTTTAT<br>CAGAATCTGAAGCTAAATCCATGGCTCAAAGACCTGGAGTTATCTCCGTATTTCCTGATCCA<br>GTACTGCAACTCCACACTACACATTCATGGGATTTCTTGAAGTATCAAACTGATGAAAAAAT<br>CAATTCAAGTCCAAGCTCTGGTTCTGATTCATCATTAATTGGAGCTGATACCATAATTGGCAT<br>ATTGGATACGGGTATATGGCCAGAATCTGAGAGTTTCAATGACAAGGATATGGGTCCAATTC<br>CATCCCGGTGGAATGGAACTTGCATGGATGGTCAAGATTTTGGCTCTTCAAAATGCAACAAG<br>AAGATAGTTGGTGCAAGATTTTATGAGGAGTCTGATGACAGTGGAACAAAAATCGCTGGATC<br>AGCCAGGGACGAGAACGGACATGGTACTCATGTTGCGTCTACTGCAGCTGGGAGTCCTGTTG<br>CAGGTGCATCCTACTATGGCCTAGCTGCAGGAACTGCCACGGGTGGATCTCCTGGTTCGAGG<br>ATTTCCATGTATCGTGTCTGTACTACTTTTGGATGCCGCGGATCAGCTATCATGAAAGCATTC<br>GATGATGCAATTGCAGATGGGGTTGATGTTTTATCACTATCACTTGGTTCATCACCTGGACTT<br>GAACCTGATTTTCCAAGCAATCCTATTGCCATAGGAGCATTTCATGCTGTAGAAAAGGGCAT<br>TACTGTTGTTTGCTCTGCTGGAAATAGTGGCCCTGGACCAAAAACTGTTGTCAATACAGCTCC<br>TTGGATTCTTACTGTTGCAGCCACCACCATTGATCGTGACTTCGAGACAGATATTGTCTTGGG<br>TGGAAACAAGCTTATTAAGGGTGGAGGTATAAACTTTGGTAACATGACAAAATCGTCAGTCT<br>ACCCTTTGATTCATGGCAATTCAACCAAATCAAACGATAATGTTTCTGAGGCGGATGCAAGG<br>AGTTGTGTTCCTGGTTCATTAGATGAAAACAAAGTCAAGGGGAAGATTGTCCTTTGTGAAAA<br>TCTTGATGATGGTGAATATTTTCCCAGTGACAAGCTAGATGAAGTGAAAAGCCGAGGTGGAG<br>TTGGATTTATACTTATAGATGATGATGAAAGAACTGTGGCACCCAAATTCAATTCCTTCTCAG<br>CTGGTGTAGTCTCTAAAAAGGATGGAAATGAGATCCTCGCCTACATTAACTCGACGAGGAAT<br>CCAGTTGCATCAATTTTACCAACTGTATCCATAACAAAGTACAAACCAGCTCCAGTTGTGGC<br>TTACTTCTCATCAAGAGGCCCTGCGTACAACACCCCTAACCTCCTCAAACCGGATATTACAG<br>CACCAGGGGTTGCCATTCTTGCTGCTTGGCCTGGAAATGACACGAGTGAGGCTCTCCCCGGC<br>CAAAAACCACCAATTTTCAACCTACTCTCAGGCACTTCCATGTCCTGCCCTCATGTATCCGGT<br>ATTGCTGCTACTGTCAAAGCAGTAAACCCTACCTGGAGTCCTTCAGCTGTCAAATCAGCTATT<br>ATGACCACAGCTATTCAGACAAACAATTTGAAGGCTCCAATCACTACAGTCTCAGGATCCAA<br>AGCAACACCATATGACATAGGTGCAGGAGAAGCAAGCACTTCAGGTCCATTAAAACCAGGT<br>CTAGTCTACGAGACAGATGTCGCCGACTACTTGCAGTTCCTATGTTCTGTTGGTTTTAACATA<br>TCACAGATAAAGCTGATCTCAATTACAGTTCCTGAAGACTTTTCATGCCCAAAAAACTCAAC<br>CTCTGAATTGGTTTCTAATATGAATTATCCATCAATAGCTATTTCTAGTCTCAAAGAAAACGA<br>GCCGAAGAAAGTTACTAGAACTGTAACAAATACTGGTGAAGAAGCATCAGTATATACTACA<br>GTTATTGAGGCACCAAAAGGATTGGAAGTCCAAGTGATCCCAACTAAATTGGAATTTACAAA<br>TAAAAGCAAGAAATTAAGCTATGATGTGTCTTTCAAAGCTTCATCTACCTCAAAGGAAGATC<br>TGTTTGGATCAATTACTTGGACTAATGGTAAGTACAAAGTCCGGAGTCCATTCGTCGTAAGT<br>AGCAACCATCATCACCATCACCACTGA

Table S2 Used plasmids.

**Plasmid**	**Description**	**Reference**
pJK155	pET28b-T7::OmpA-His-TEV-EpiC1	This work
pCC03	Bacterial CapB expression	This work
pCC04	Bacterial CspD expression	This work
pJK082	pL1V-pET28b	Kourelis et al., 2020
pJP001	pL0M-PSU-T7-LacO-T7 transl Strong RBS	Kourelis et al., 2020
pJP002	pL0M-3UT-T7 terminator	Kourelis et al., 2020
pJK120	pL0M0NT2-OmpA-6xHIS6-TEV	Kourelis et al., 2020
pFGH027	Binary EpiC1	Grosse-Holz et al., 2018
pJK001c	Binary EV control	Paulus et al., 2020
pJP008	Binary P69B-His	Paulus et al., 2020
pFGH048	Binary Epi1	Grosse-Holz et al., 2018
pPB097	Binary SBT5.2a-His	This work
TRV::GUS	*TRV::GUS* control	Duggan et al., 2021
pPB039	*TRV::SBT5.2*	Beritza et al., 2024
pID024	*TRV::CORE*	Dodds et al., 2023
TRV2::PDS	*TRV::PDS*	Liu et al., 2002
TRV1	RNA1 of TRV	Liu et al., 2002
pJK001c	pL1V2-F1; cloning vector	Paulus et al., 2020
pICH51288	pL0M-P5U-2x35S-TMV	Engler and Marillonnet, 2014
pICH41414	pL0M-3UT-T35S	Engler and Marillonnet, 2014

Table S3 Used synthetic peptides.

Peptide	Description	Manufacturer
LNGKVKWFNNAKGYGFILEDGK	csp22 (CspD)	Genscript
Dabcyl-VKWFNNAK-Edans	Qcsp8	Genscript
DTGTVKWFNTSKGFGFISRDSG	csp22 PSPTO3984	Genscript
QTGTVKWFNDEKGFGFITPQSG	csp22 PSPTO4145	Genscript
