# Supplementary figures and images for: Extracellular plant subtilases dampen cold-shock peptide elicitor levels
Source: Nat Plants. 2024 Oct 11;10(11):1749–60. doi: 10.1038/s41477-024-01815-8 (PMC11570497; doi:10.1038/s41477-024-01815-8)

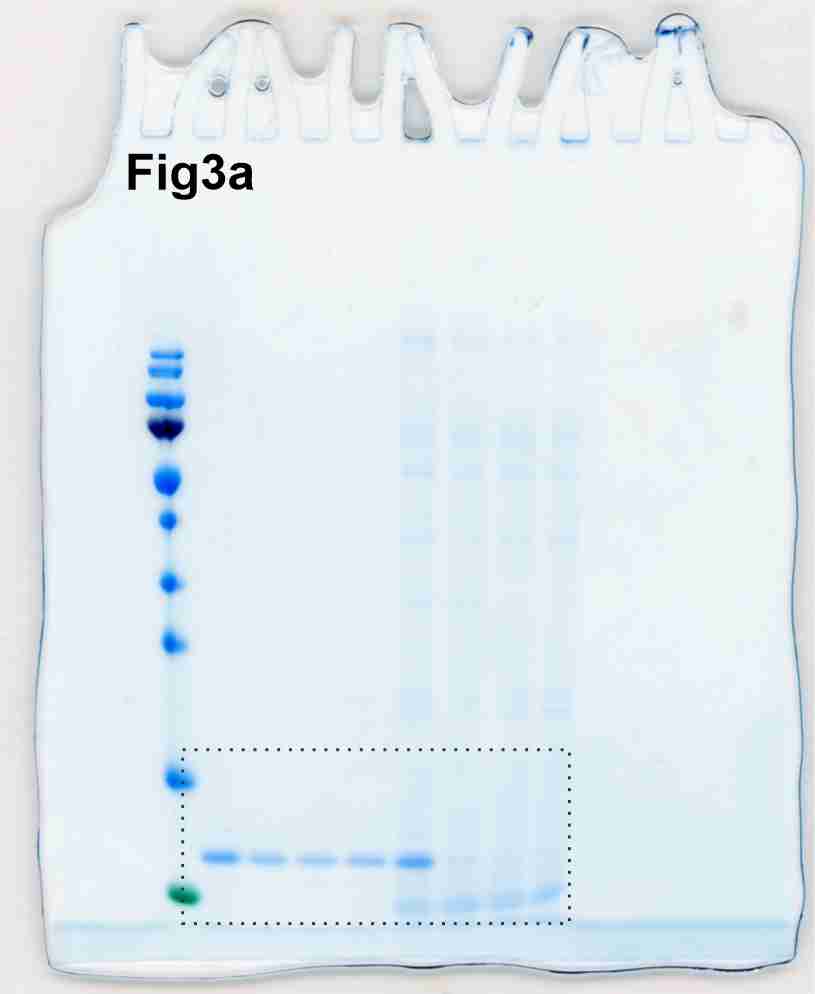

Supplement: Supplementary file 3 — Uncropped gel for Fig. 3a. [file 41477_2024_1815_MOESM3_ESM.jpg]

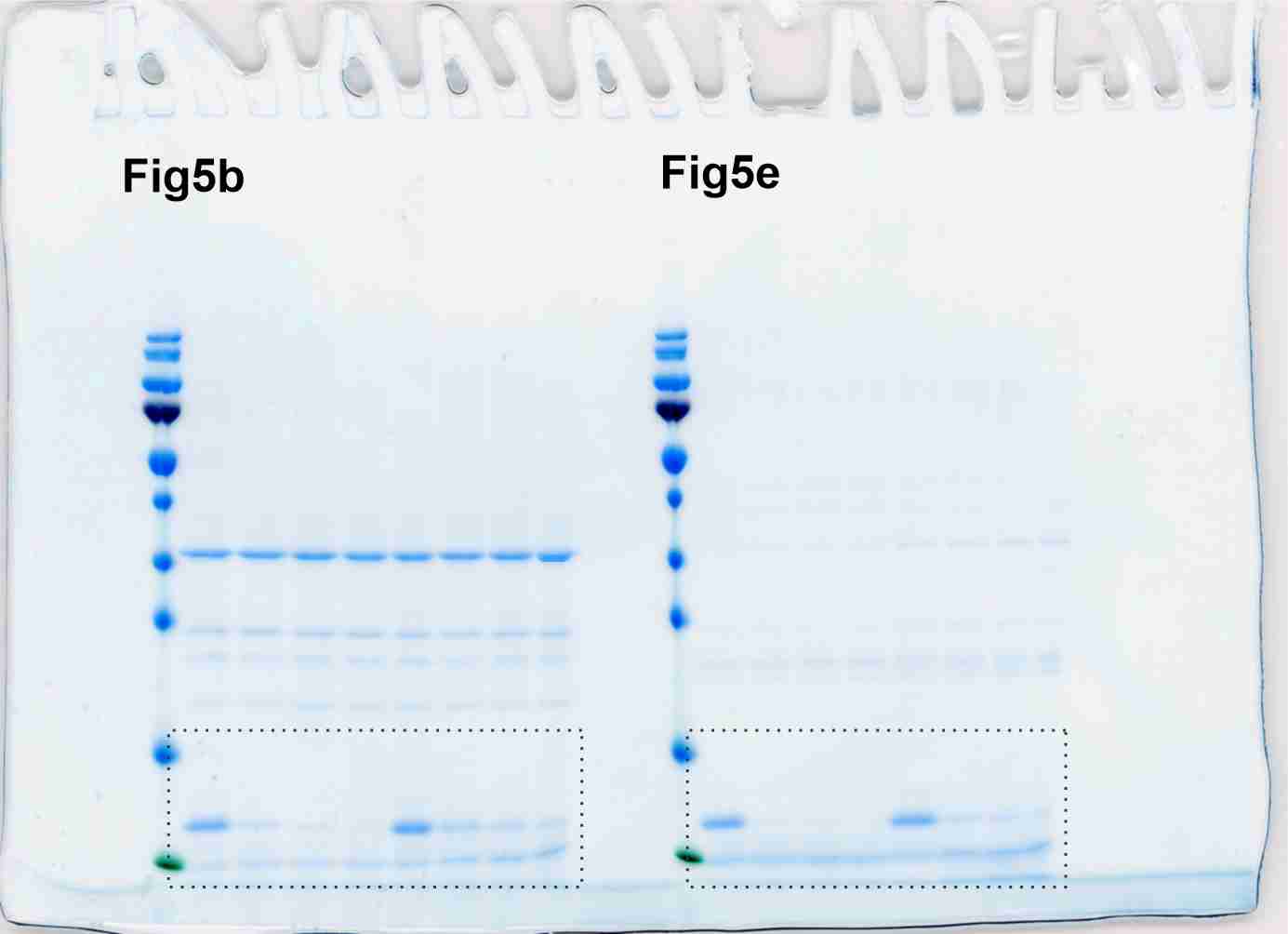

Supplement: Supplementary file 4 — Uncropped gel for Fig. 5b,e. [file 41477_2024_1815_MOESM4_ESM.jpg]

**Fig6a**

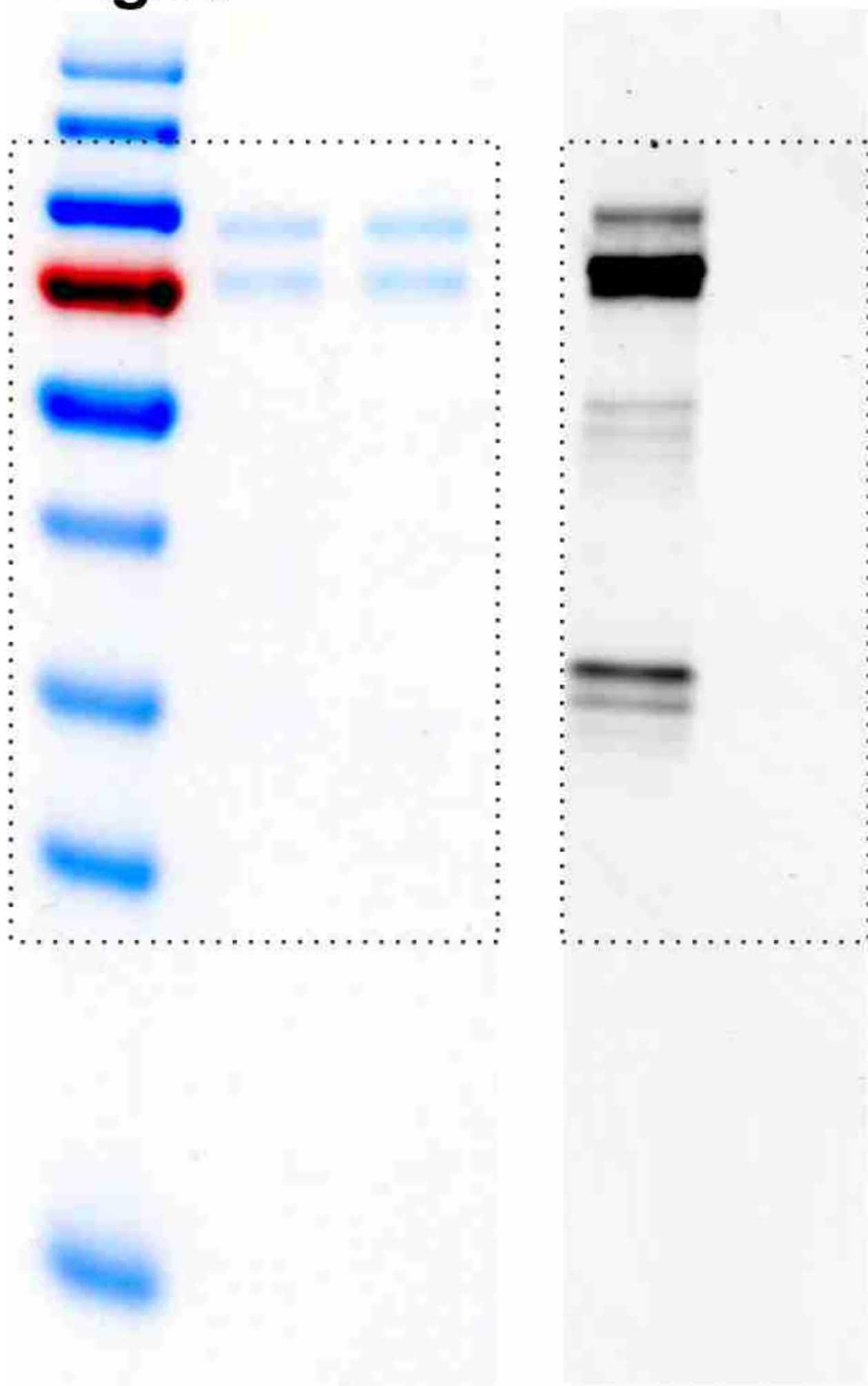

**Fig6c**

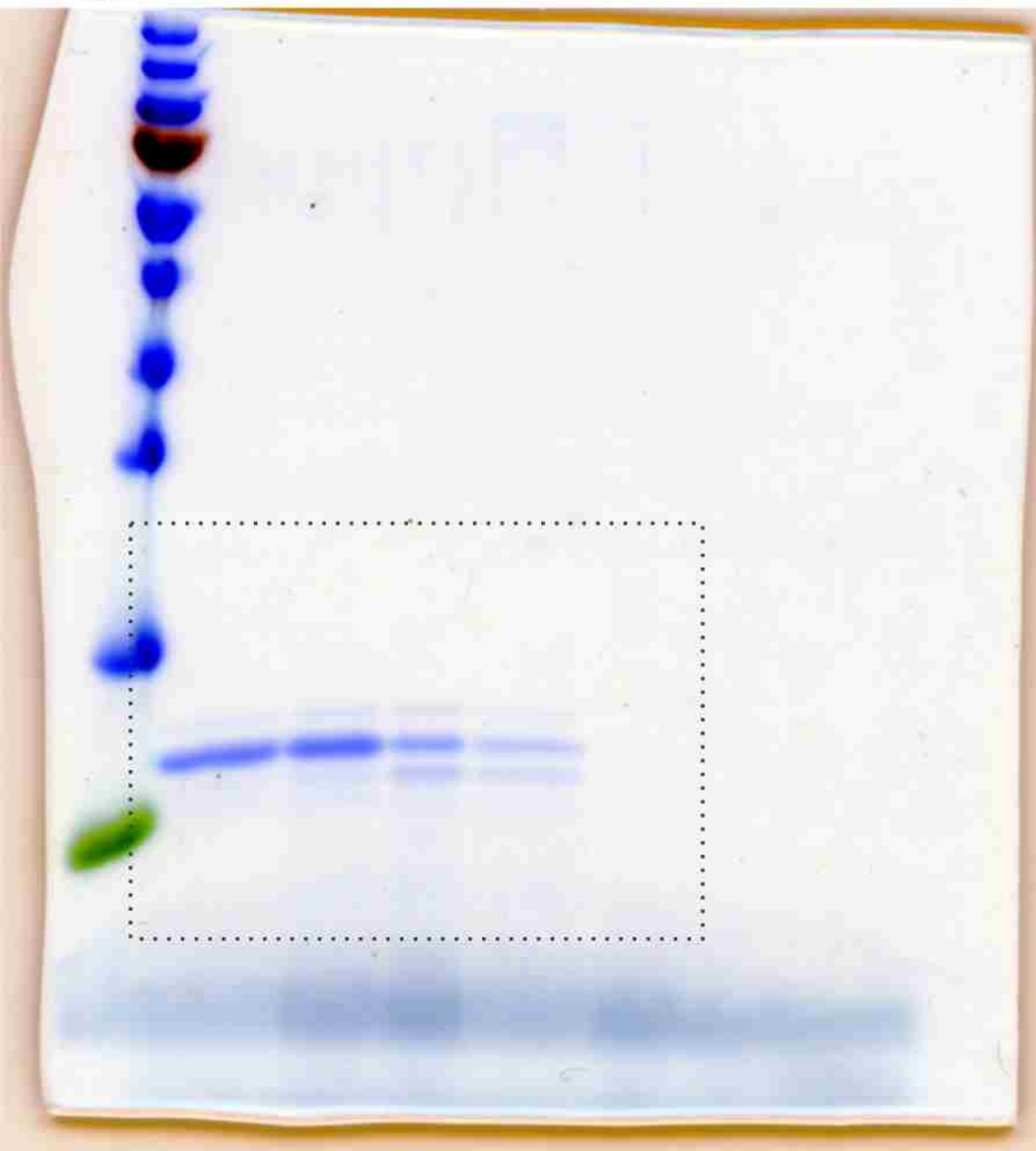

Supplement: Supplementary file 5 — Uncropped gel for Fig. 6a,c. [file 41477_2024_1815_MOESM5_ESM.pdf]
